# The impact of clinical nutrition on cancer therapy: a frequently underestimated perspective. A complementary approach to cancer patients

**DOI:** 10.1007/s12349-012-0105-z

**Published:** 2012-08-05

**Authors:** Samir Giuseppe Sukkar, The ONCONUT® Project Group

**Affiliations:** U.O., Dietetica e Nutrizione Clinica, IRCCS Az. Ospedaliera Universitaria san Martino-IST di Genova Università di Genova, Pad. VII piano 2, Largo R. Benzi 10, 16132 Genoa, Italy

Since the onset of the disease, oncologic patients frequently show a weight loss that varies according to cancer location, type and stage [1]. The neoplastic diseases that mostly induce malnutrition are Hodgkin’s lymphoma, gastrointestinal cancer, and head and neck tumors [2].

Inversely related to prognosis, malnutrition causes a worsening in the quality of life, with an increase of morbidity and mortality [3, 4]. In a reasonable percentage of neoplastic patients, the first cause of death seems to be due more to a metabolic-nutritional unbalance than to the disease itself [5].

In a recent French trial in 879 patients, Pressoir et al. [6] has found that a moderate to severe malnutrition is related with extended periods of hospitalization: 19.3 ± 19.4 vs. 13.3 ± 19.4 days (*P* < 0.001).

In 2010, Platek et al. [7] demonstrated that in patients with head and neck tumors, who were undergoing an antiblastic treatment, >10 % pretreatment weight loss was related with an incomplete locoregional response.

Moreover, in the univariate analysis, malnourished patients showed a worsened *physical function* (*P* = 0.007) and an increased *fatigue* (*P* = 0.034) compared to well-nourished patients; in the multivariate analysis, instead, malnutrition was significantly related to *physical function* (*P* = 0.015) [8].

It is known, indeed, that the cytokine unbalances are already present during the initial diagnosis and continue over time, leading to the so-called cachexia–anorexia syndrome, i.e. the tumor–host interaction that determines, in this disorder as well as in many others, the patient’s progressive malnutrition.

The nutritional pathway must therefore consider the tumors in their unitary aspect, in order to continuously take care of the patient, from the diagnosis to the acute and chronic consequences of the oncologic therapy, until the desirable recovery or up to the terminal phase.

According to ASPEN guidelines, it is necessary to evaluate patient nutritional status, since the beginning of the diagnostic–therapeutic course, carefully controlling it throughout the treatment, and changing it when specific needs are required [9].

A survey conducted by CERGAS-Bocconi in 787 patients of 110 Cancer Treatment Centers has pointed out that oncologists poorly understand the discomfort of patients who are unable to communicate it, and they mostly take into consideration symptoms like nausea (62 %), pain (53 %) instead of depression, frequently ignored (15 %), and fatigue (30 %).

Malnutrition in cancer patients is defined as neoplastic cachexia–anorexia syndrome and is characterized by weight loss, adynamia, anorexia, hyporexia and asthenia. This condition shows a multifactorial pathogenesis including food intake reduction (often associated with appetite and taste changes, disease-related diet depletion, and psychological distress), tumor–host interaction (through tumoral cachexia-inducing substances, neuroendocrine mechanisms and cytokine network changes) and side effects caused by anti-cancer treatments [chemotherapy (CT), radiotherapy (RT), surgery]. As a result, changes in energy, protein, lipid and glucose metabolism are observed.

For this reason, a failed adaptation of neoplastic patients marks the changes in the body composition, with extracellular mass expansion, cellular and fat mass reduction, as well as energy loss.

The nutritional status of neoplastic patients is directly related to chemotherapy tolerability, therefore to its efficacy, and to patient’s quality of life. A questionnaire providing a correct nutritional assessment, and the patient biological findings enable the planning of a correct diet. This strategy allows the improvement in chemotherapy tolerability and patient’s quality of life.

Based on such considerations, the need of a multidisciplinary approach in the management of onco-hematological patients arises, through the collaboration of onco-hematologists, nutritionists, psychologists, chemists, nurses, dietitian, as well as social workers and physiotherapists.

The nutritional assessment of cancer patients includes a careful history (medical, nutritional and pharmacological), physical examination with anthropometric measurements (present weight, usual weight, weight loss during the last 6 months, upper arm muscle circumference, triceps skinfold), laboratory test (albumin, total proteins, transferrin, prealbumin, lymphocytes), functional test (dynamometry) and caloric consumption test, with possible measure of physical activity level (PAL), that corresponds to the total energy expenditure/resting energy expenditure (TEE/REE) ratio.

Finally, considering a holistic-nutritional approach, the assessment of quality of life is essential (by means of specific questionnaires like EORTC, SPITZER and TQ).

All these outcomes can be collected in indexes, where a defined score is assigned to each parameter. The sum or the product derived will enable to classify malnutrition in a specific grading (mild, moderate, severe).

Among these, most utilized and validated items are Subjective Global Assessment (SGA), Patient-generated SGA (PG-SGA), MUST and Nutritional Risk Index (NRI).

## Are the outcomes related to nutritional therapy evaluated? If so, in which way?

The nutritional intervention strategy in cancer patients obviously implies a suitable initial evaluation through a screening that defines the malnutrition severity. According to malnutrition staging, the patient will be inserted in a specific therapeutic and diagnostic program.

In case of normal nutritional status, the patient will be monitored each month; in case of mild malnutrition, the patient will undergo further nutritional assessment and dietetic history, as to allow a tailored nutritional counseling; in case of moderate–severe malnutrition, the patient will be treated with multimodal therapies (anti-anorexic agents, dietetic/artificial integration, prokinetic and antiemetic drugs). In order to evaluate the nutritional support efficacy in cancer patients, anthropometric, biochemical, functional and metabolic data should be analyzed, as well as performance status, quality of life and psychological findings.

For instance, one of the primary outcomes is the evaluation of body weight; however, it is important that the weight gain reflects the increase of metabolically active tissue. For this reason, the simple quantitative assessment of body weight can be misleading, because it does not assess the body qualitative composition (i.e. fat mass increase versus lean one) or the hydration status of patients (presence of ascites or edema).

Also the metabolic status have to be evaluated: patient with cancer show an increased REE; this depends on tumor mass, cancer type, chemotherapy drugs, disease duration and patient nutritional status. Published trials have shown that nutritional supply with certain substances can increase TEE/REE ratio, improving PAL.

Another important finding is the determination of performance status: the Karnofsky index (KI) measures, in percentage from 0 to 100, the patient’s capability to carry out daily activities and the probable need of assistance support.

Also quality of life, certainly influenced by body weight, is evaluated, since the progressive neoplastic cachexia can lead to depression, asthenia and anxiety, with relevant loss in appetite and caloric intake (useful assessment instruments are EORTC-ALA-30, Spitzer index, TIQ (Therapy Impact Questionnaire).

Finally MUST, PG-SGA, SGA assessments scales are utilized, since they are useful till the onset of disease, as first nutritional screening methods, and after, as evaluation tools of re-nutrition effects.

## The fatigue

The fatigue is the most common chronic symptom in cancer patients.

An exact definition of fatigue does not exist, since common notion of tiredness sometimes overlaps the clinically relevant symptom of fatigue. It can be defined as the difficulty to start and sustain voluntary activities [10].

The peripheral fatigue can be clinically distinguished from the central fatigue. The first defines the muscle fatigue due to alterations of muscles or of neuromuscular junction; it is objectively defined, measured by the force peak decline rate produced during the maximum voluntary muscle contraction and it is evaluated by electromyography.

Peripheral fatigue is typically observed in case of myasthenia, metabolic myopathies, mitochondrial myopathies and hypothyroidism.

Central fatigue shows a feeling of constant exhaustion. Its severity is independent of the nature and severity of underlying diseases (like MS, Parkinson’s disease, migraine, mitochondrial diseases, etc.), and it undergoes periodic fluctuations in relation to different psychological or physiological stimuli. In a study conducted by Ashbury et al. [11], 913 patients were treated with antiblastic therapy for 2 years; the fatigue, reported in 78 % of patients, worsened the normal daily activities in 71 % of the cases. In patients with metastatic cancer, fatigue is present in more than 75 % of cases, and finally, according to Hopwood (in a multicenter, randomized study in patients with lung cancer, aged 39–90 years), fatigue and weakness were observed in >80 % of patients. Fatigue, therefore, involves all stages of the disease and can persist even several months after the accomplishment of the therapy and considerably after recovery. This element is common to the other form of pathological fatigue: chronic fatigue syndrome (CFS). Cancer-related fatigue (CRF) results in the worsening of patients’ quality of life, can cause treatment discontinuation, and may increase health care management costs. However, fatigue is often poorly considered by clinicians, whose attention is mainly focused on pain and disease-free survival, despite the significant impact of fatigue on patient’s quality of life. In patients older than 70 years, the fatigue is present in 70–99 % of cases.

In 144 treated patients with cancer of lung, esophagus, stomach and pancreas (Table 1), very recent data from our group (ONCONUT^®^ project), which evaluated the impact of Whey protein (Prother^®^—Spepharm Italia) supplement on fatigue in cancer patients, show a low positive correlation between fatigue and weight loss (*r* = 0.224) (Table 2) [12].

**Table 1:** Baseline characteristics of a sample of 144 cancer patients undergoing treatment (lung 68, stomach 30, esophagus 12, pancreas 31) [12]

	Mean	Standard deviation	Median	Percentile 25	Percentile 75	*N* (%)
Age	68	10	70	63	76	
Usual weight	72	15	70	62	83	
Present weight	66.9	14.6	64.8	56.9	76.0	
%Weight loss	6.166	8.389	4.430	0.122	11.911	
Height (m)	1.68	0.11	1.69	1.61	1.73	
BMI	23.6	4.4	23.0	20.5	26.2	
CMB (cm)	26.5	4.2	27.0	24.0	29.0	
Triceps skinfold (cm)	12.4	7.3	10.0	7.0	14.4	
hgs dx	26	9	25	20	32	
hgs sn	25	9	25	18	30	
Karnofsky index						
40						1 (0.7)
60						2 (1.4)
70						20 (13.9)
80						47 (32.6)
90						48 (33.3)
100						26 (18.1)
Fatigue	6.055	2.067	6.255	4.550	7.590	
VAS appetite	6	3	6	4	8	
VAS nausea	2	3	0	0	5	
VAS pain	3	3	3	0	5	
Blood glucose (mg/dl)	126	48	109	96	141	
Creatinine (mg/dl)	1.3	5.1	0.8	0.7	1.0	
Blood urea (mg/dl)	39.17	15.73	35.00	29.00	45.00	
Tot. proteins (g/l)	49.830	28.260	63.000	7.250	69.000	
Albumin (g/l)	27.87	12.33	33.00	25.00	36.00	
Transferrin (mg/dl)	235.111	55.797	228.000	202.000	251.000	
Cholinesterase (UI/ml)	6,208.58	1,985.47	6,100.00	4,800.00	7,300.00	
CPR	13.39	19.40	6.20	1.90	12.30	
Lymphocytes (mm^3^)	2.04	2.33	1.74	1.24	2.28	
REE kcal tot (HB)	1,770	253	1,728	1,600	1,900	
Prot requirements	82	16	82	71	91	
kcal intake	1,459	434	1,434	1,183	1,711	
Prot intake	69.2	90.7	60.0	46.0	74.2	
Hydric intake	1,029.02	535.35	1,000.00	500.00	1,500.00	

*Hgs* hand grip strength

**Table 2:** Correlations between fatigue, weight loss, appetite, pain and Karnofsky status in 144 cancer patients in therapeutic phase [12]

Patients (*n* = 144)	Fatigue	%Weight loss	VAS appetite	VAS nausea	VAS pain	Karnofsky
Fatigue						
Pearson		0.224	−0.139	0.238	0.354	−0.462
*P* value		0.007	0.097	0.004	0.000	0.000
%Weight loss						
Pearson			−0.081	0.000	0.066	−0.526
*P* value			0.332	1.000	0.434	0.000
VAS appetite						
Pearson				−0.130	−0.152	0.172
*P* value				0.122	0.069	0.039
VAS nausea						
Pearson					0.408	−0.233
*P* value					0.000	0.005
VAS pain						
Pearson						−0.321
*P* value						0.000

A significant inverse relationship exists between fatigue and Karnofsky status (*r* = −0.46, *P* < 0.001): when one value increases, the other one decreases, and vice versa.

A positive, but low correlation, and therefore a weak direct relationship, exists between fatigue and pain (*r* = 0.354, *P* < 0.001).

The clinical nutrition represents a supportive element to cancer treatment, and its goal, synergistic to it, is to reach the cure.

Therefore, in cancer patients, the therapeutic nutrition course reproduces the specific clinical intervention and integrates with it in a constant over time continuum, in the light of typical therapeutic goals of oncology: survival, symptoms control and improved quality of life. Considering the future therapeutic strategies in oncology and the relevant goals in terms of quality of life, it is mandatory not only to seek even more effective and “smart” chemotherapies, but also to limit the incidence of related toxic effects, by a more accurate study of oxidative stress and substances able to antagonize its consequences.

According to the general project, the “parallel metabolic-nutritional approach”, as suggested by Muscaritoli et al. [13] activated in cancer patients, must be structured in parallel to the anti-neoplastic treatment, and the early involvement of a dietitian in the oncology team offers the advantage of an easy and rapid contact with patients referring to the oncological structure.

The nutritional intervention, according to the ONCONUT^®^ Group pathway [12], must be articulated through:

- carrying out a nutritional screening in order to assess the risk of malnutrition,
- history of weight and possible weight loss,
- careful history of usual intake of food protein–calories, and 24 h recall,
- evaluation of present coverage percentage versus calculated protein–calories requirements,
- detection of symptoms linked to food ingestion and to any gastrointestinal disorder,
- execution of functional tests: hand grip strength evaluation,
- assessment of fatigue, quality of life and performance status.

Information and strategies must be given to patients to be able to feed properly and adequately. Indeed, the literature has demonstrated that the dietary counseling, when compared with a diet ad libitum or with nutritional supplementation, is able to improve the prognosis and the QoL of cancer patients [14].

If the simple counseling is not enough to achieve the requirements, the goal of the clinical nutritionist is to plan a more complete nutritional assessment in dietetics and clinical nutrition structures, for a more articulated nutritional intervention, with possible prescription of artificial oral supplementation, and/or activation of enteral and/or parenteral nutrition.

## References

[1] Bozzetti F, Migliavacca S, Scotti A (1982). Impact of cancer type, site and treatment on the nutritional status of patients. Ann Surg.

[2] De Wys WE, Bogg C, Lavin PP (1980). Prognostic effect of weight loss prior to chemotherapy in cancer patients. Am J Med.

[3] De Wys WE, Daly JM, Dudrick SJ, Copeland EM (1979). Evaluation of nutritional indices as prognostic indicators in the cancer patients. Cancer.

[4] Von Meyenfeldt MF, Fedrix EW, Haagh WA (1988). The aetiology and management of weight loss and malnutrition in cancer patients. Baillieres Clin Gastroenterol.

[5] Hinagaki J, Rodriguez V, Bodey GP (1974). Cause of death in cancer patient. Cancer.

[6] Pressoir M, Desné S (2010). Prevalence, risk factors and clinical implications of malnutrition in French Comprehensive Cancer Centres. Br J Cancer.

[7] Platek ME, Reid ME et al (2011) Pretreatment nutritional status and locoregional failure of patients with head and neck cancer undergoing definitive concurrent chemoradiation therapy. Head Neck 33:1561–1568 10.1002/hed.21640 PMC4403643 21990220

[8] Harriet J-W (2011). Malnutrition in patients treated for oral or oropharyngeal cancer—prevalence and relationship with oral symptoms: an explorative study. Support Care Cancer.

[9] Arends J, Bodoky G, Bozzetti F (2006). ESPEN Guidelines on Enteral Nutrition: non-surgical oncology. Clin Nutr.

[10] Chaudhuri A, Behan PO (2004) Fatigue in neurological disorders. Lancet 363:978–988 10.1016/S0140-6736(04)15794-2 15043967

[11] Ashbury FD (1998). A Canadian survey of cancer patients’ experiences: are their needs being met?. J Pain Symptom Manag.

[12] Sukkar SG, Noli G, Amerio ML, Pucciariello A, Muzio F, Semeraro M, Sabbatini A, Valoriani F, Signori A (2012) Qualità Gestionale Nutrizionale In Oncologia: Il Progetto Onconut. In Atti dell’11° Congresso Nazionale Progress in Clinical Nutrition, Senigallia

[13] Muscaritoli M, Molfino A, Gioia G, Laviano A, Rossi Fanelli F (2011). The “parallel pathway”: a novel nutritional and metabolic approach to cancer patients. Int Emerg Med.

[14] Ravasco P, Monteiro-Grillo I, Marques Vidal P, Camilo ME (2005). Impact of nutrition on outcome: a prospective randomized controlled trial in patients with head and neck cancer undergoing radiotherapy. Head Neck.

